# Supplementary material for: Meiotic drive of female-inherited supernumerary chromosomes in a pathogenic fungus
Source: eLife. 2018 Dec 13;7:e40251. doi: 10.7554/eLife.40251 (PMC6331196; doi:10.7554/eLife.40251)
Supplement: Figure 3—source data 1. [file elife-40251-fig3-data1.pdf]

A

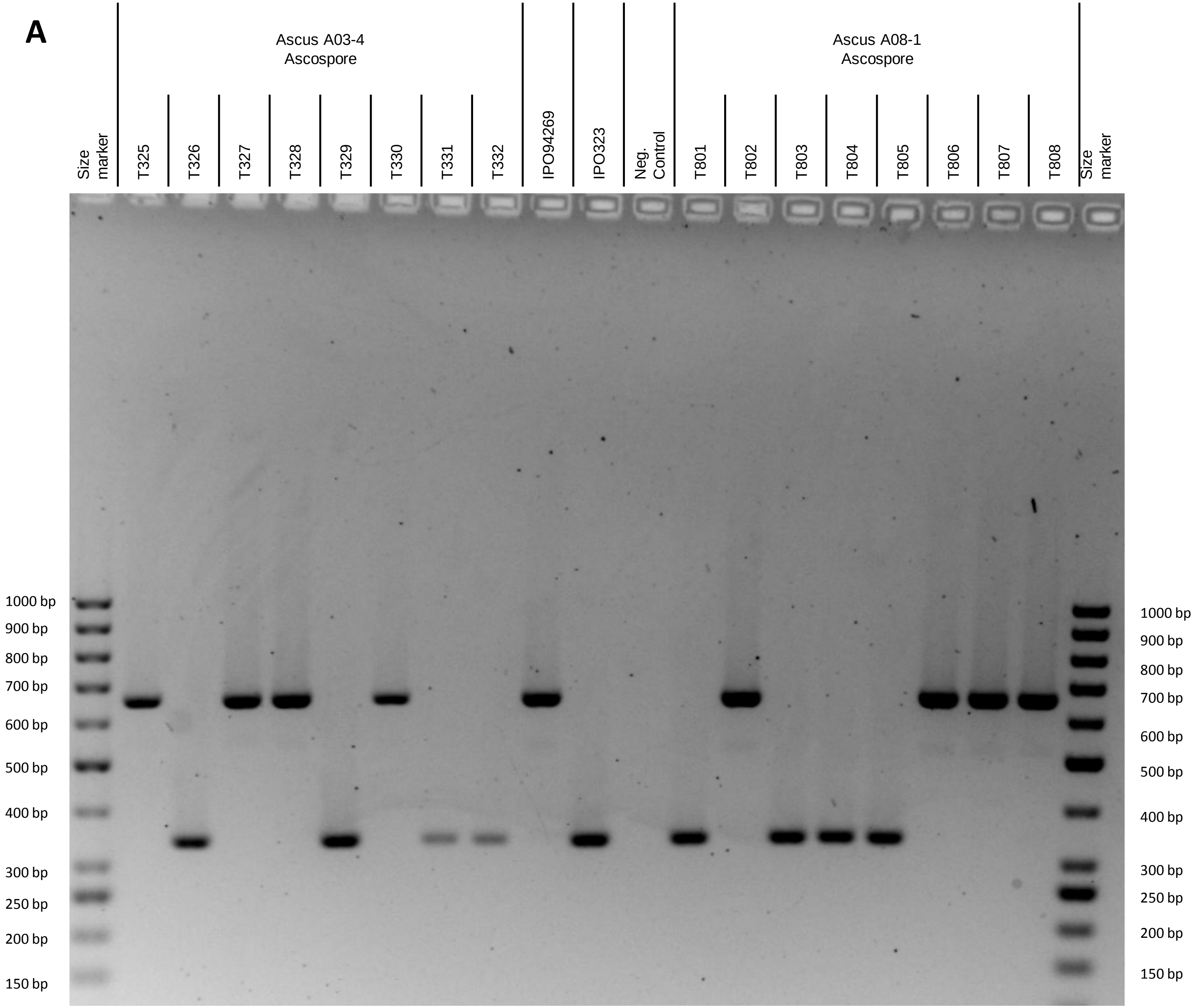

chr	Marker	Primer-No	expected product size in IPO323 [bp]	Expected product size in IPO94269 [bp]
13	Mating type	MAT1-1F<br>MAT1-1R<br>MAT1-2F<br>MAT1-2R	340	660

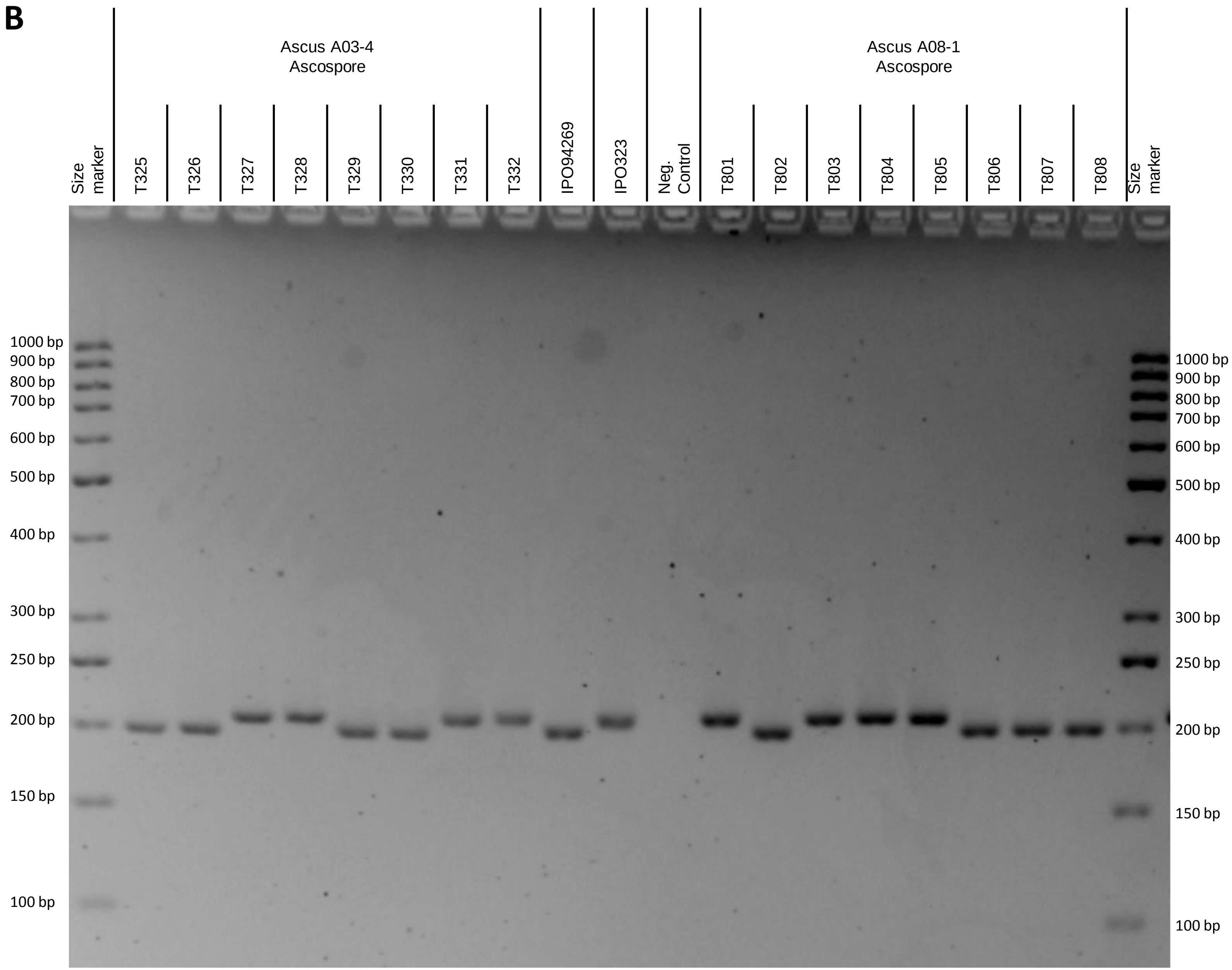

chr	Marker	Primer-No	expected product size in IPO323 [bp]	Expected product size in IPO94269 [bp]
4	11O21	11O21F 11O21R	205	199

C

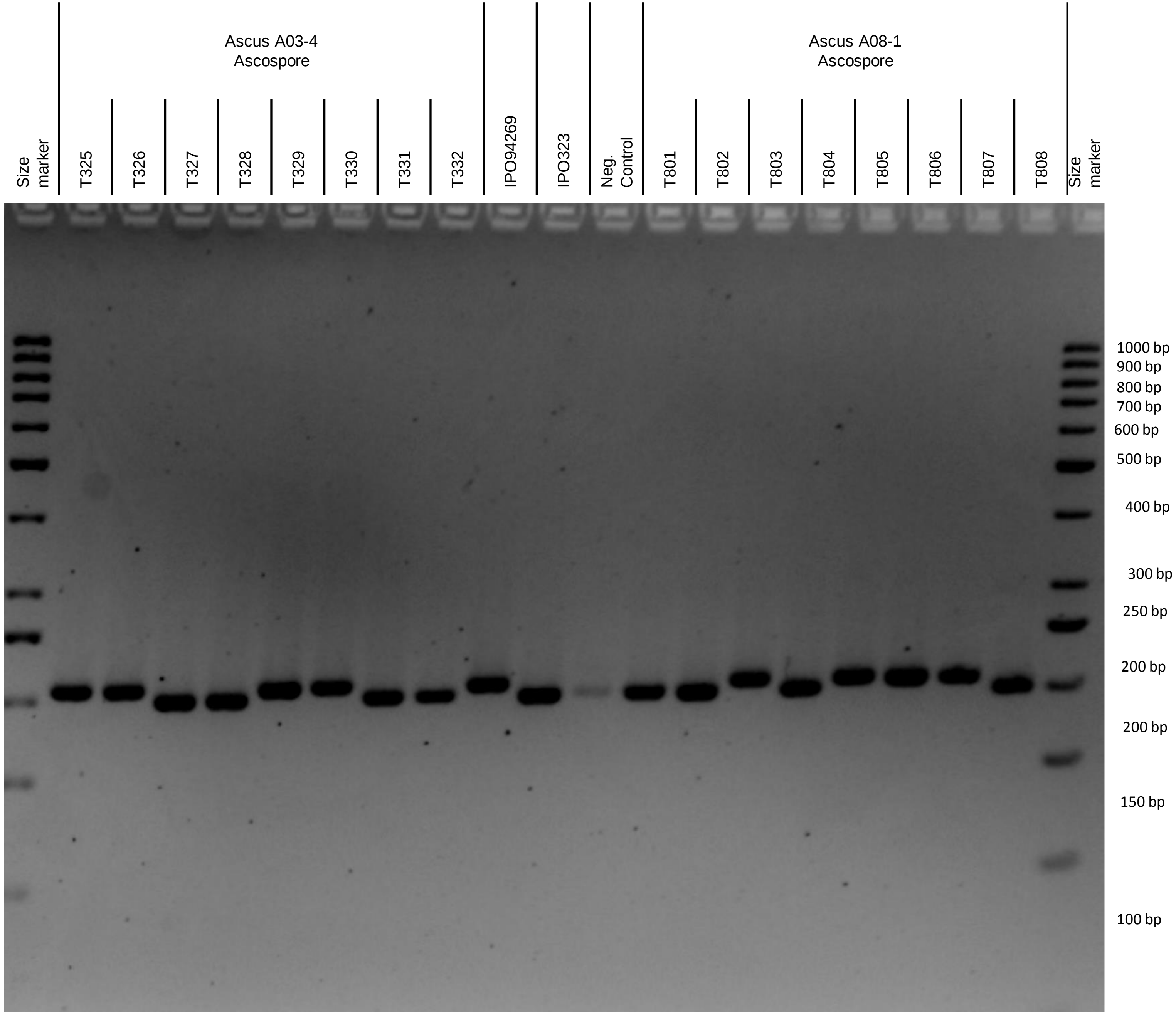

chr	Marker	Primer-No	expected product size in IPO323 [bp]	Expected product size in IPO94269 [bp]
4	04L20	04L20F 04L20R	192	199

D

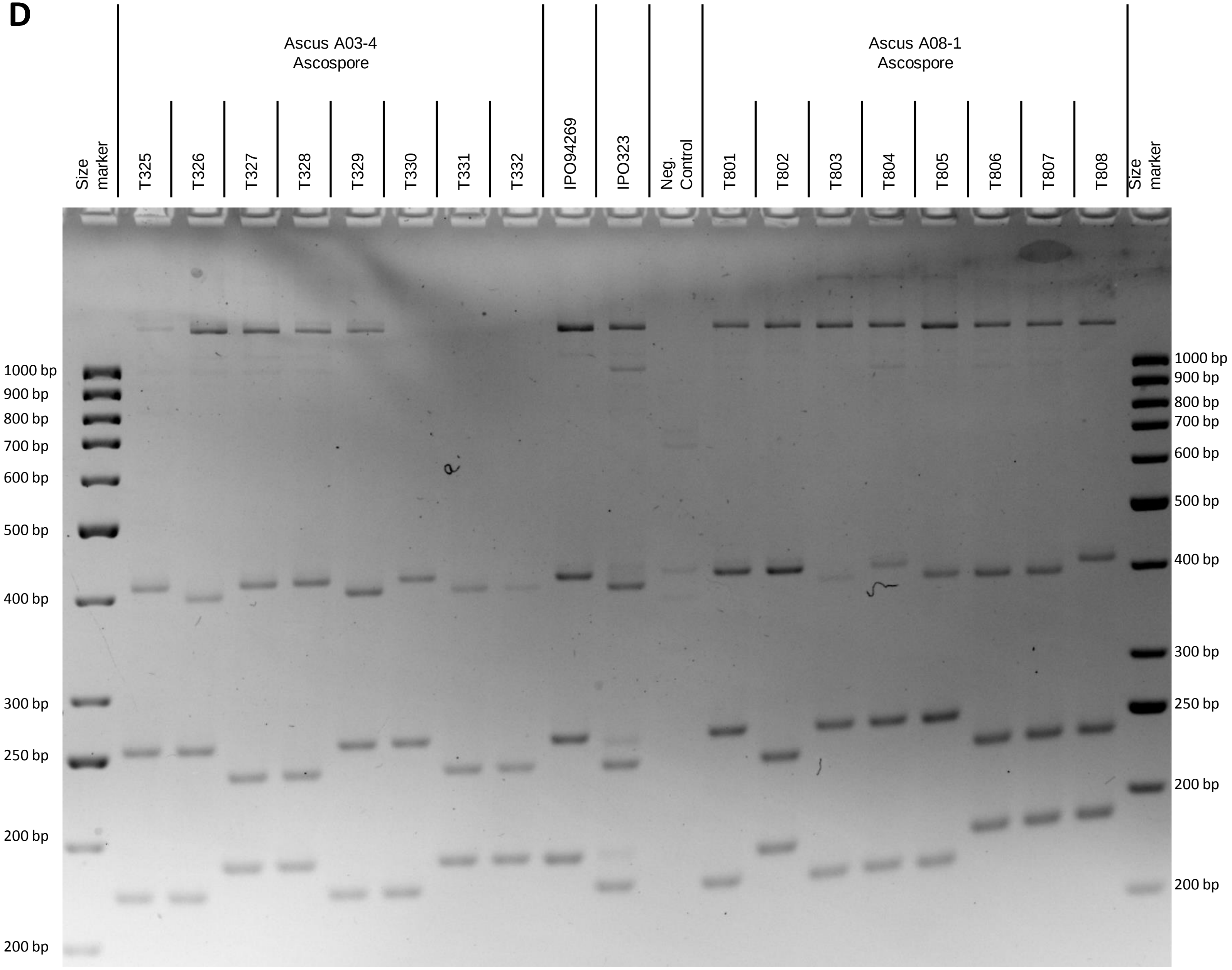

chr	Marker	Primer-No	expected product size in IPO323 [bp]	Expected product size in IPO94269 [bp]
3	caa-0002	2996*2997	412	396
5	ggc-001	2998*2999	254	234
7	ac-001	3000*3001	187	173

E

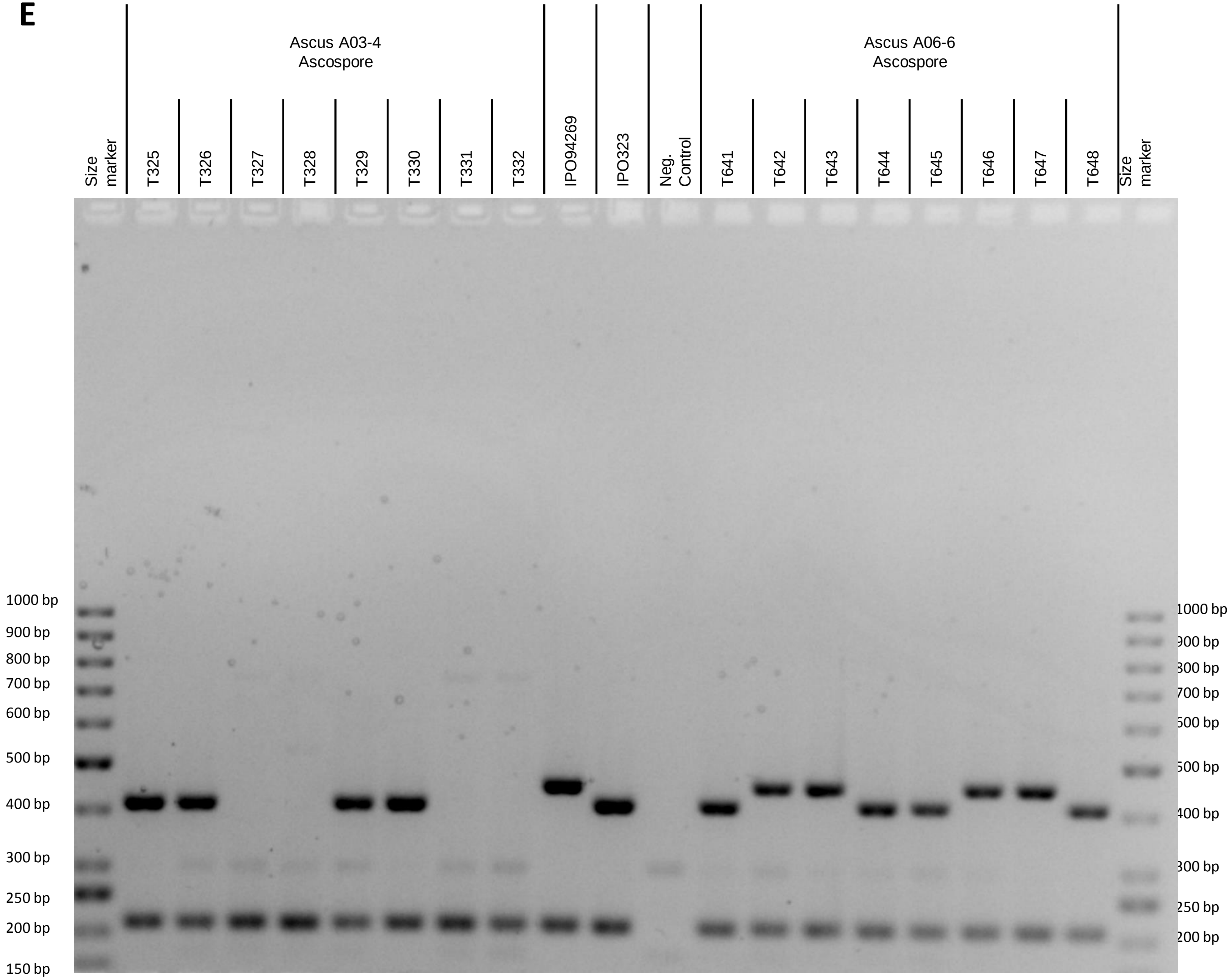

chr	Primer-No	expected product size in IPO323 [bp]	Expected product size in IPO94269 [bp]
15	3008*3009	413	511
2	879*880 (GAPDH)	207	207

F

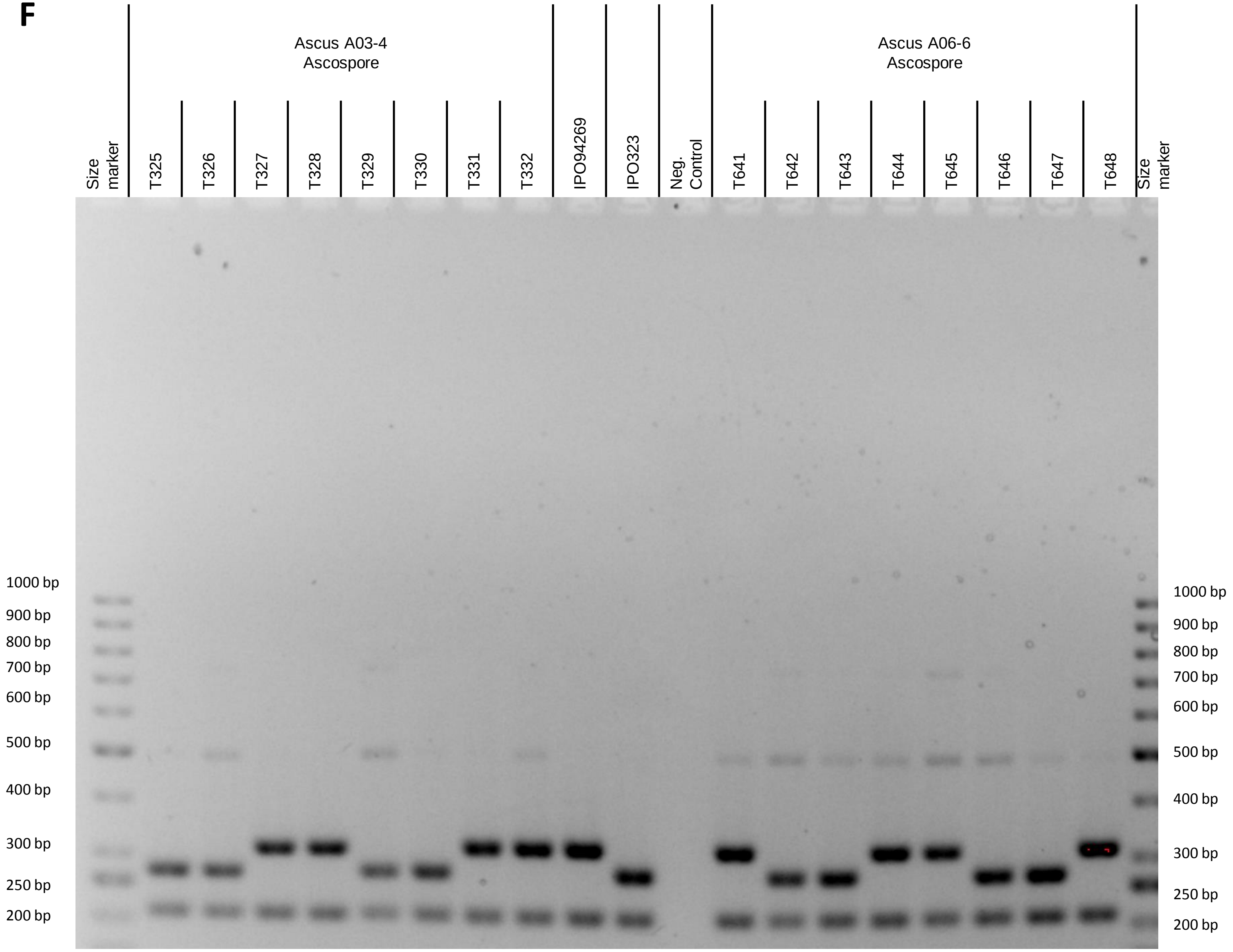

chr	Primer-No	expected product size in IPO323 [bp]	Expected product size in IPO94269 [bp]
14	3006*3007	272	312
2	879*880 (GAPDH)	207	207

G

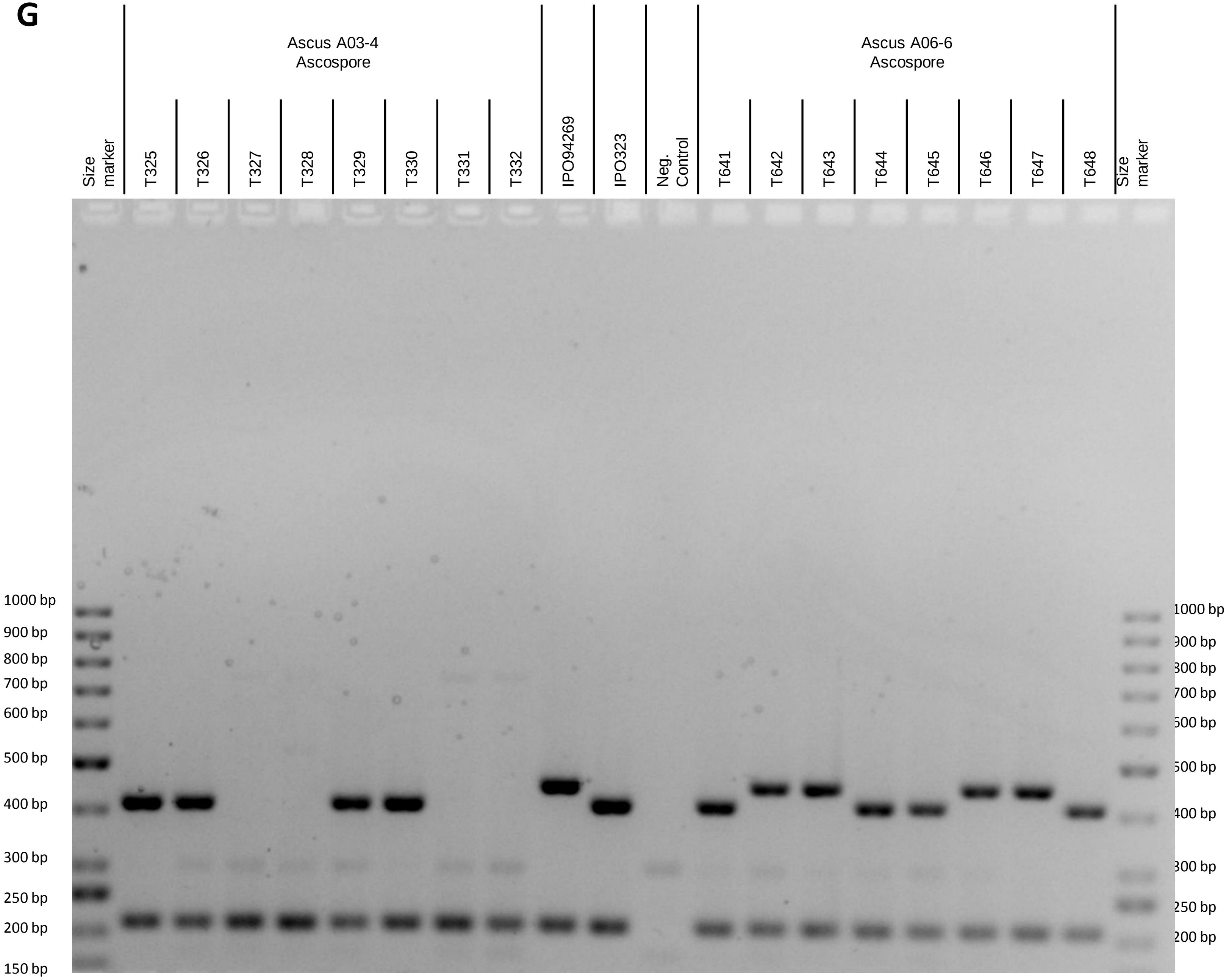

chr	Primer-No	expected product size in IPO323 [bp]	Expected product size in IPO94269 [bp]
15	3008*3009	413	511
2	879*880 (GAPDH)	207	207

H

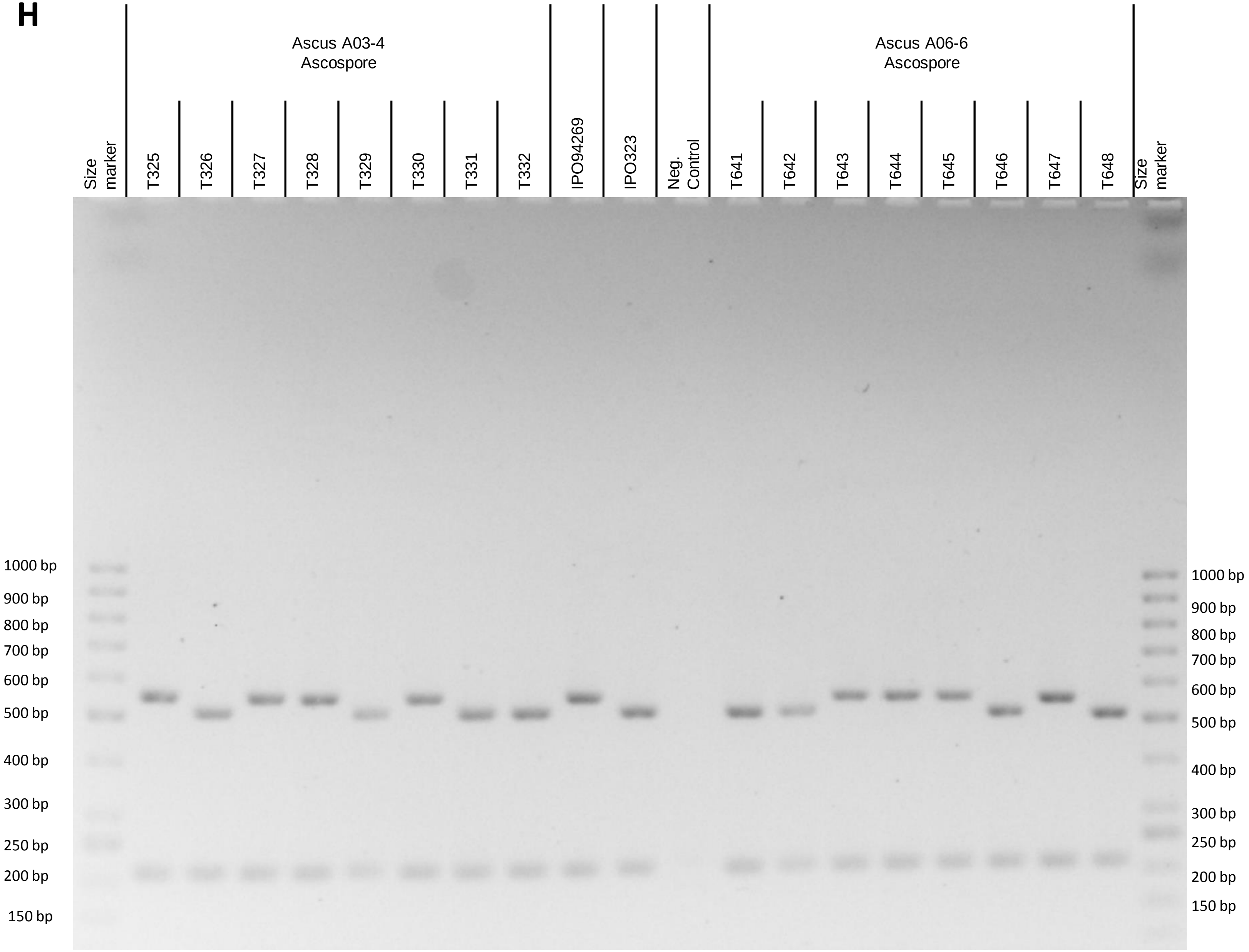

chr	Primer-No	expected product size in IPO323 [bp]	Expected product size in IPO94269 [bp]
16	3010*3011	511	549
2	879*880 (GAPDH)	207	207

I

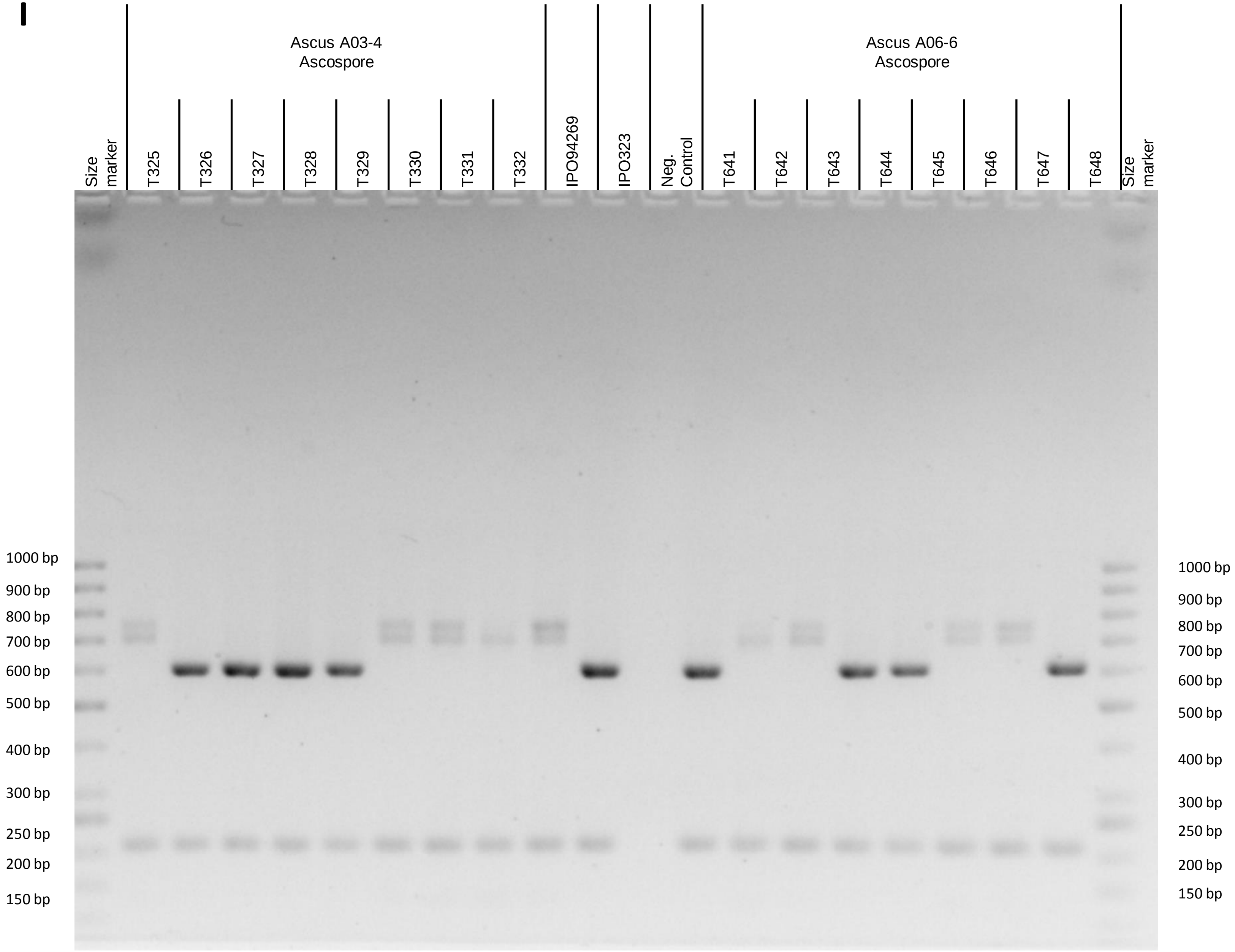

chr	Primer-No	expected product size in IPO323 [bp]	Expected product size in IPO94269 [bp]
17	3012*3013	613	745
2	879*880 (GAPDH)	207	207

J

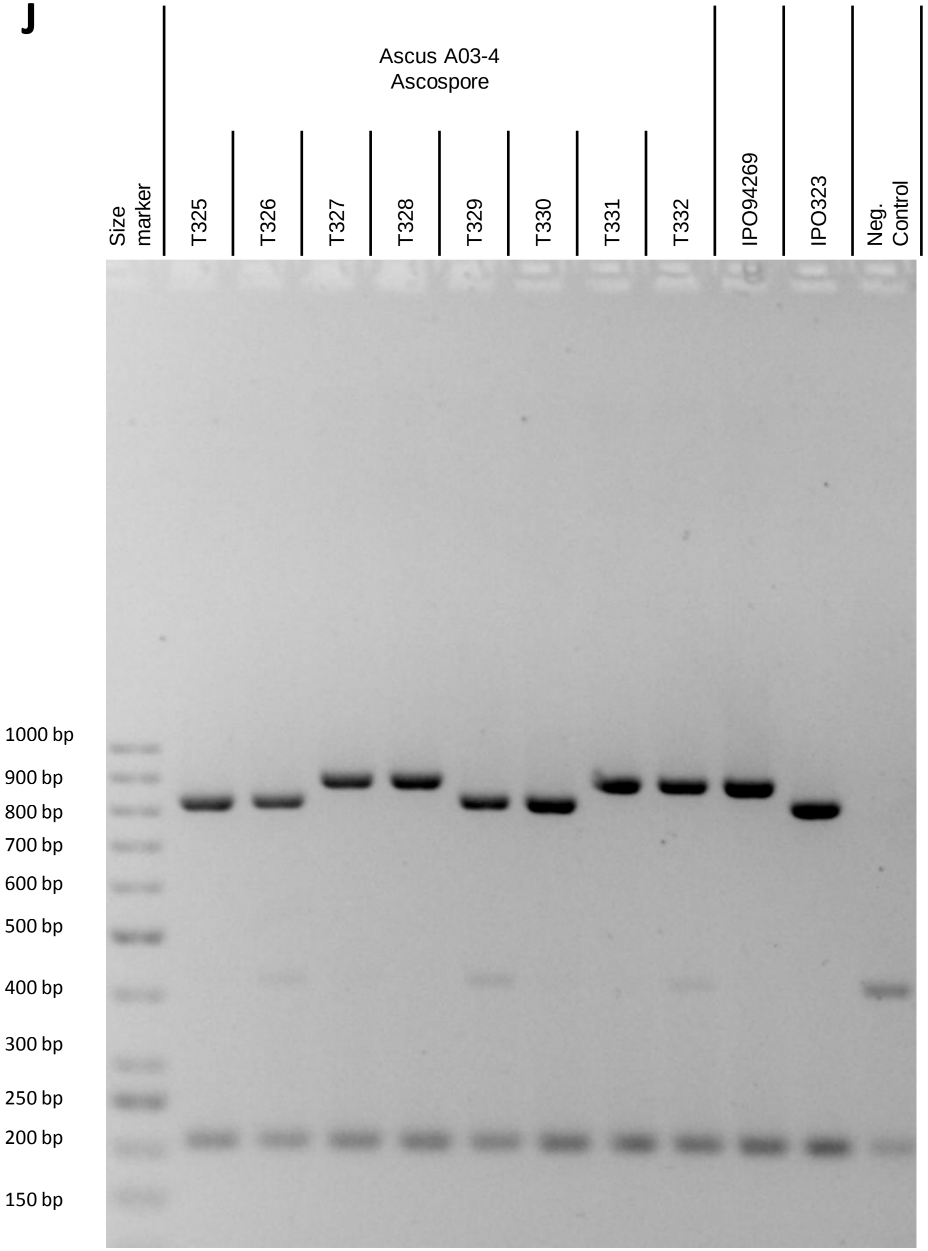

chr	Primer-No	expected product size in IPO323 [bp]	Expected product size in IPO94269 [bp]
19	3014*3015	822	886
2	879*880 (GAPDH)	207	207

K

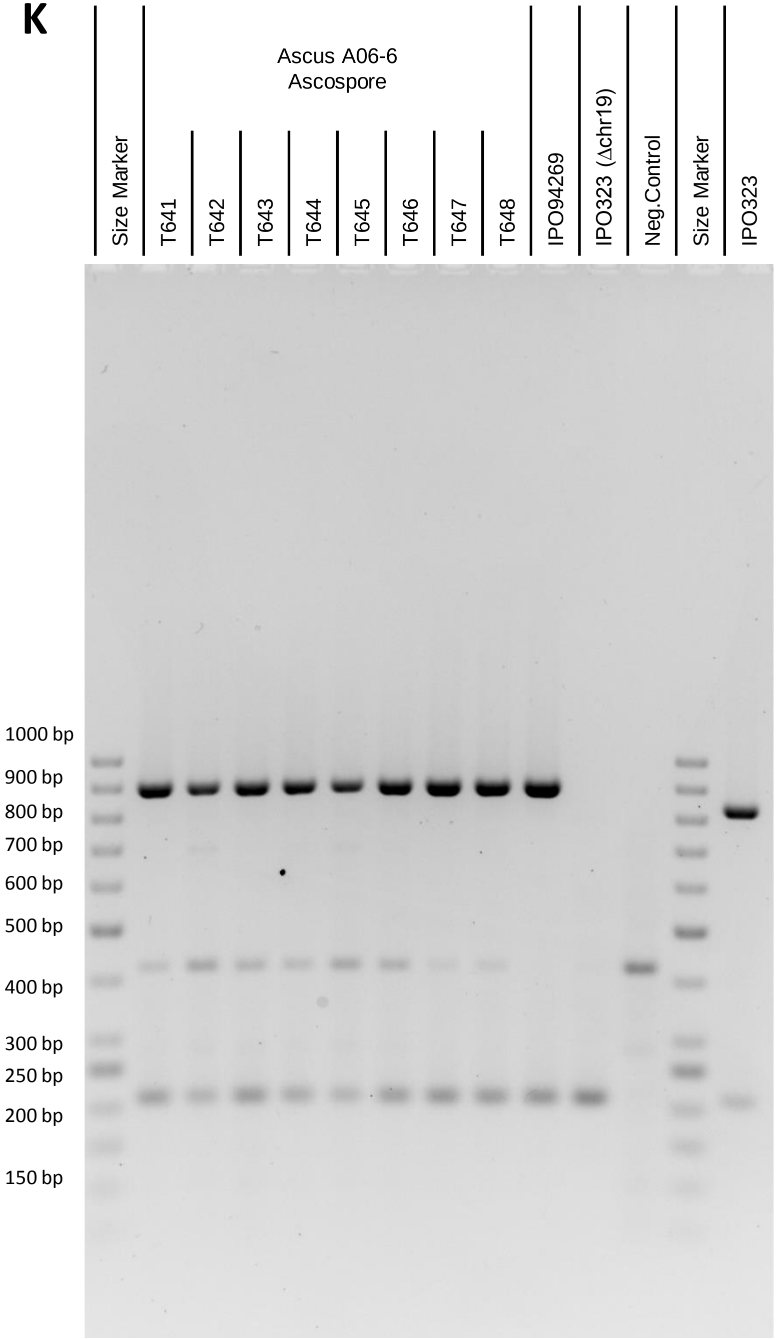

chr	Primer-No	expected product size in IPO323 [bp]	Expected product size in IPO94269 [bp]
19	3014*3015	822	886
2	879*880 (GAPDH)	207	207

L

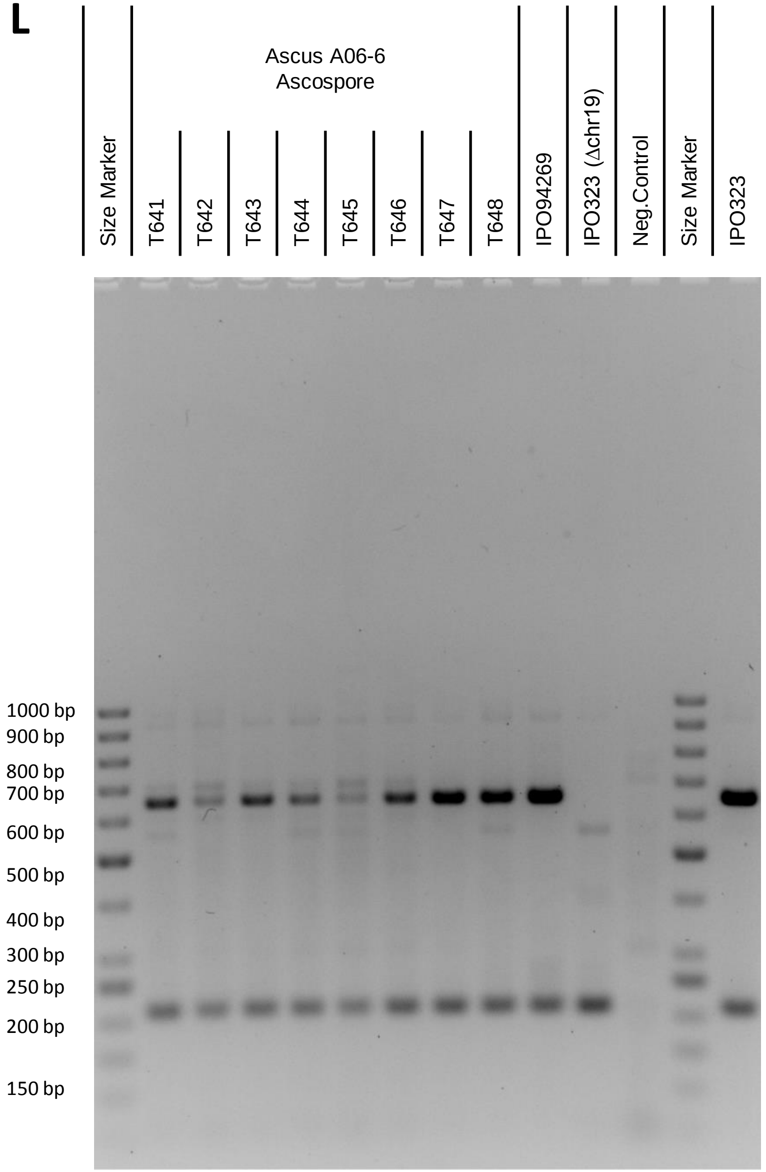

chr	Primer-No	expected product size in IPO323 [bp]	Expected product size in IPO94269 [bp]
19	2104*2105	652	652
2	879*880 (GAPDH)	207	207

M

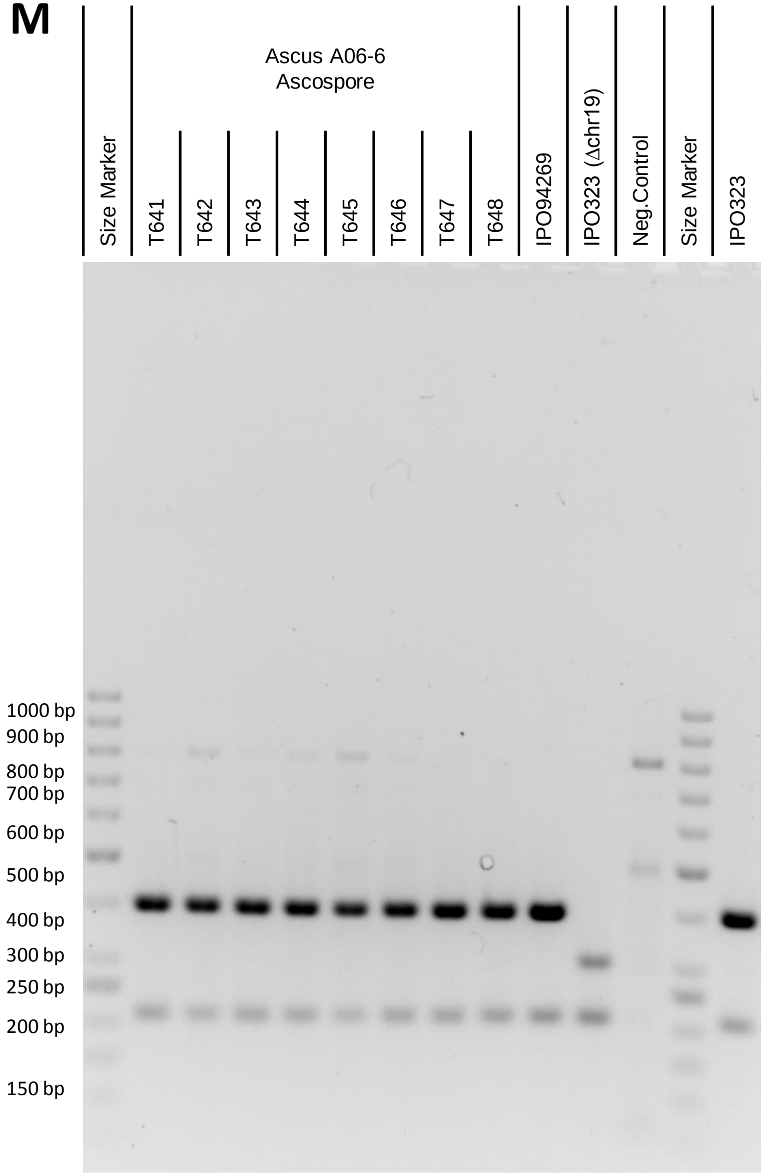

chr	Primer-No	expected product size in IPO323 [bp]	Expected product size in IPO94269 [bp]
19	3024*3025	405	405
2	879*880 (GAPDH)	207	207

N

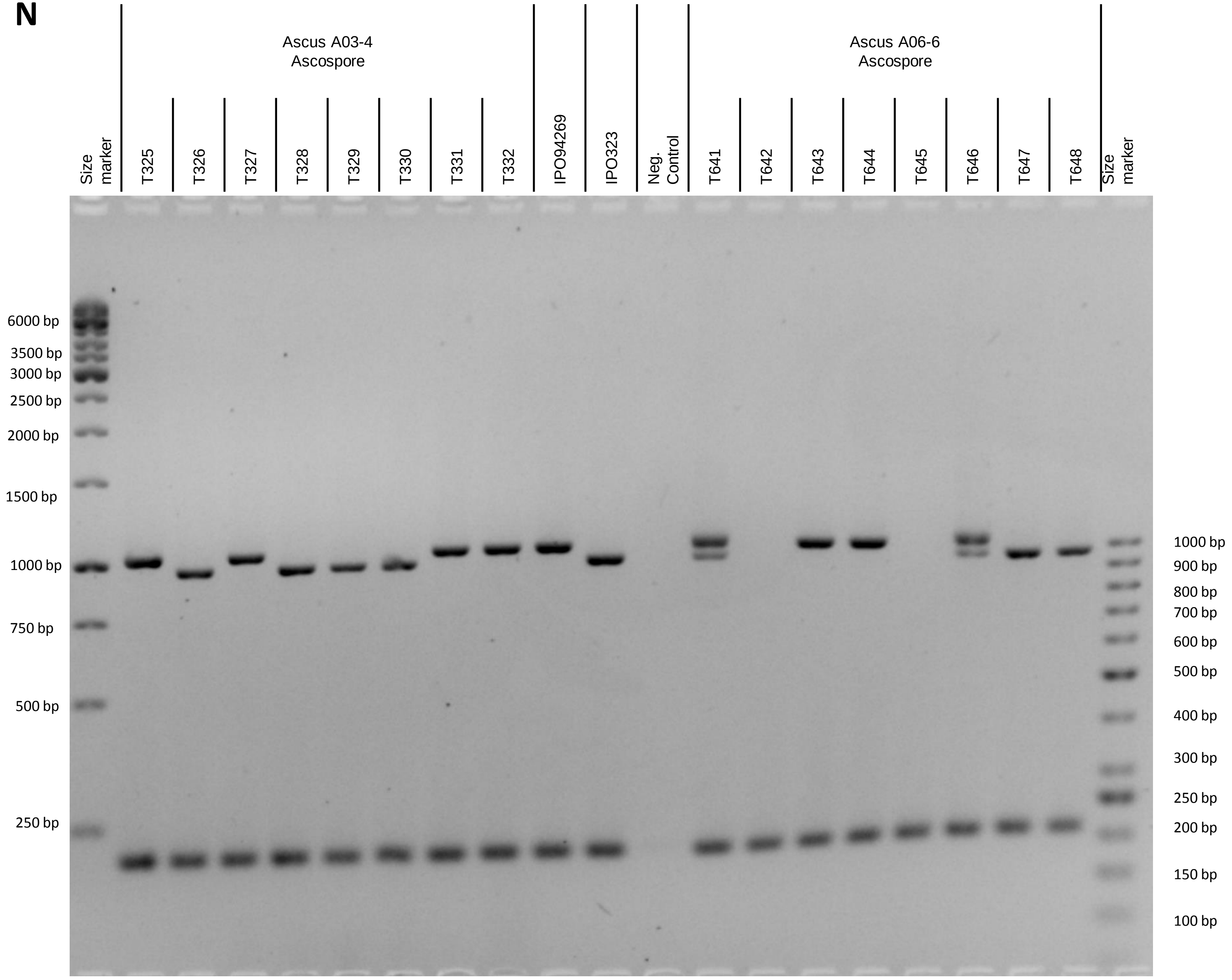

chr	Primer-No	expected product size in IPO323 [bp]	Expected product size in IPO94269 [bp]
21	3016*3017	955	1018
2	879*880 (GAPDH)	207	207

O

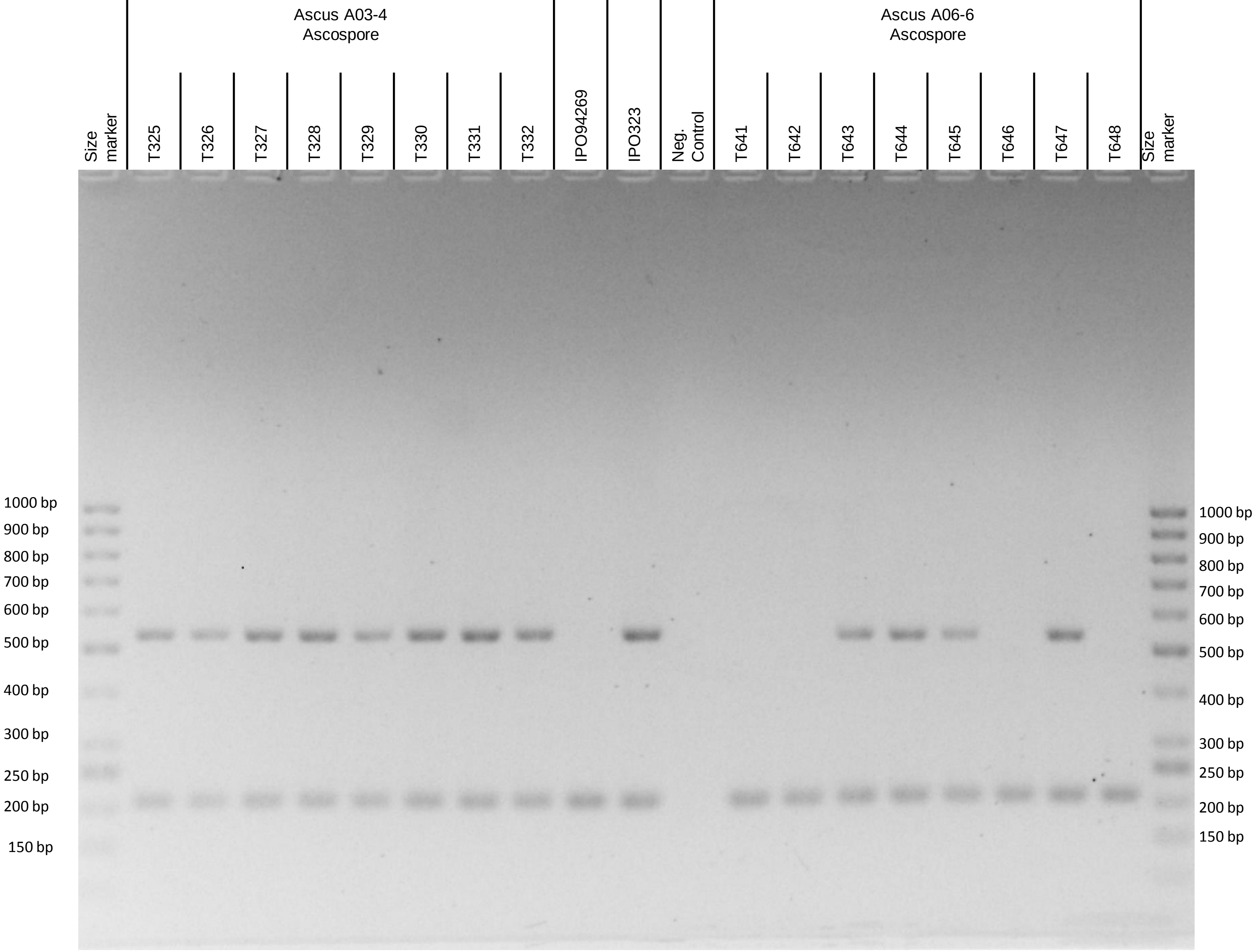

chr	Region	Primer-No	expected product size in IPO323 [bp]	Expected product size in IPO94269 [bp]
18	Subtelomeric L	2100*2101	550	-
2		879*880 (GAPDH)	207	207

P

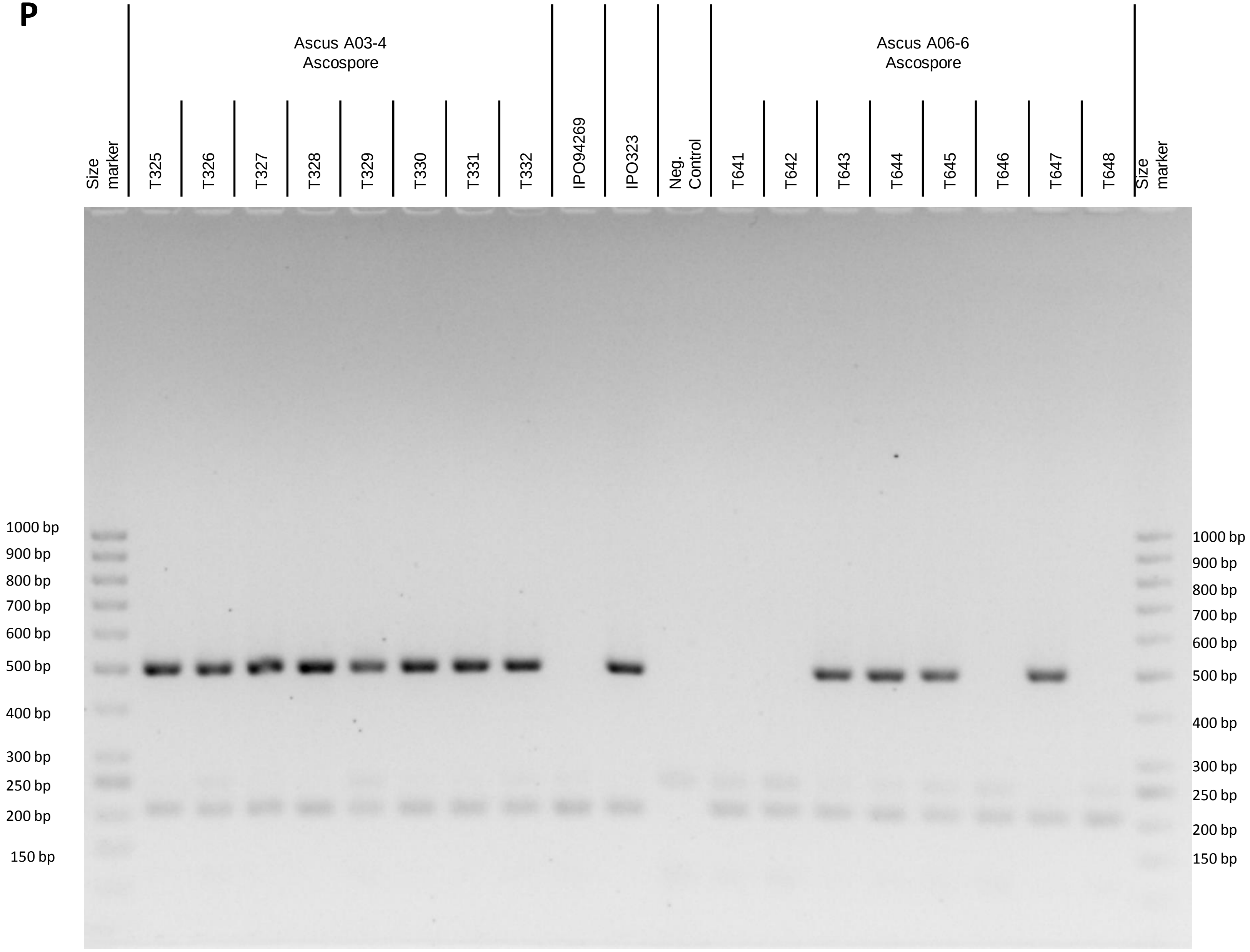

chr	Region	Primer-No	expected product size in IPO323 [bp]	Expected product size in IPO94269 [bp]
18	centromeric	2908*2909	500	-
2		879*880 (GAPDH)	207	207

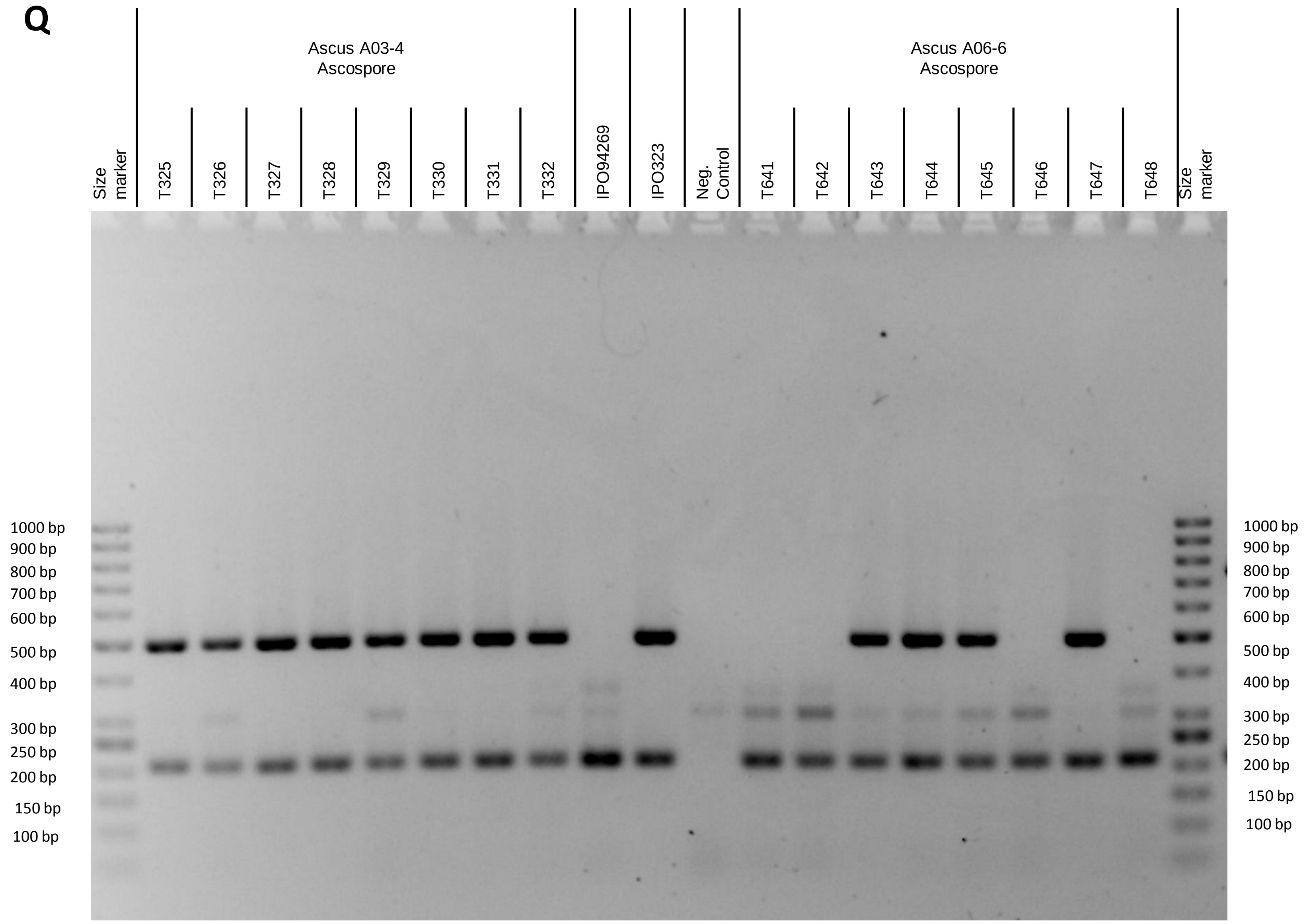

chr	Region	Primer-No	expected product size in IPO323 [bp]	Expected product size in IPO94269 [bp]
18	Subtelo mericR	2102*2103	500	-
2		879*880 (GAPDH)	207	207

R

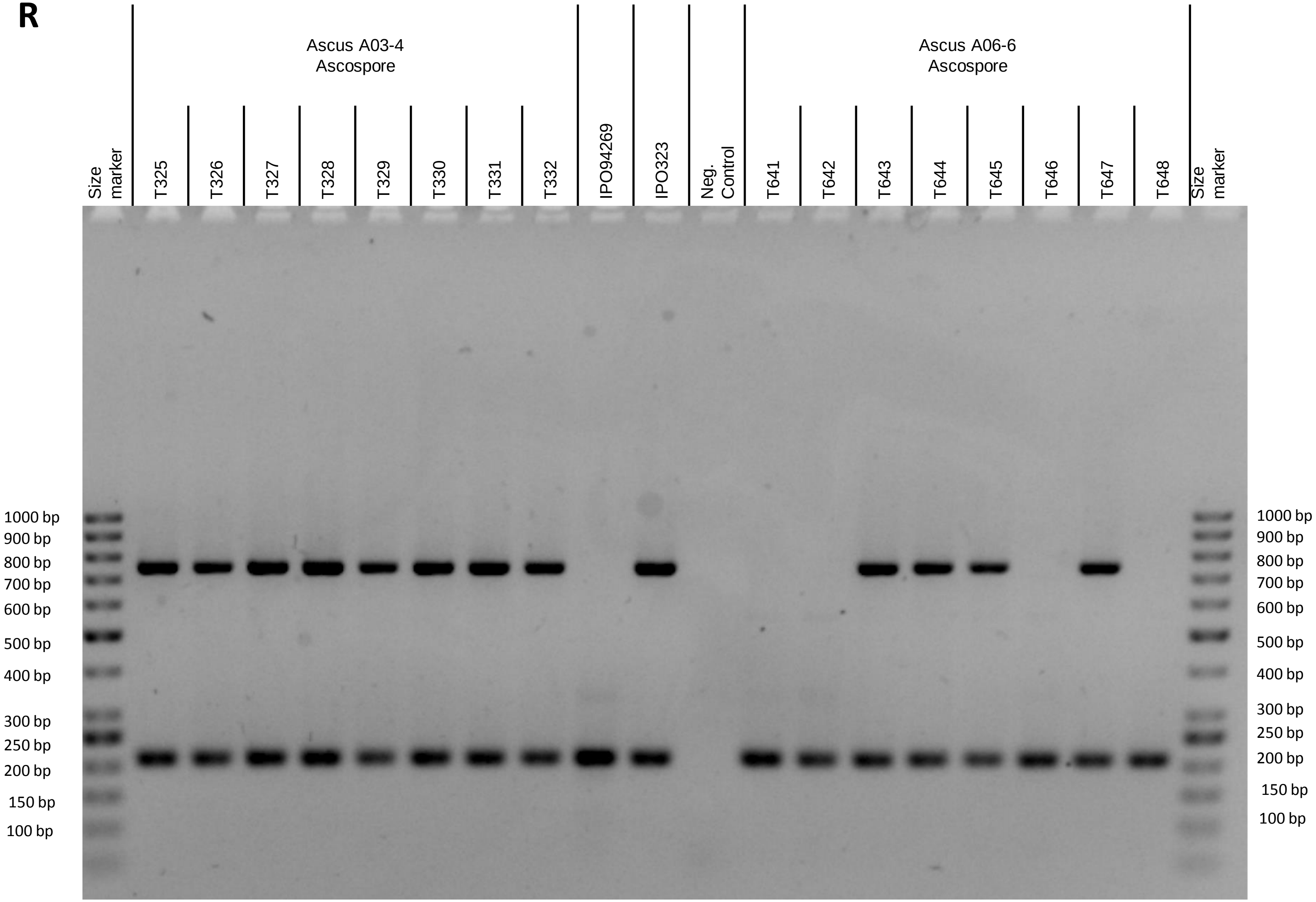

chr	Region	Primer-No	expected product size in IPO323 [bp]	Expected product size in IPO94269 [bp]
20	SubtelomericL	2108*2109	750	-
2		879*880 (GAPDH)	207	207

S

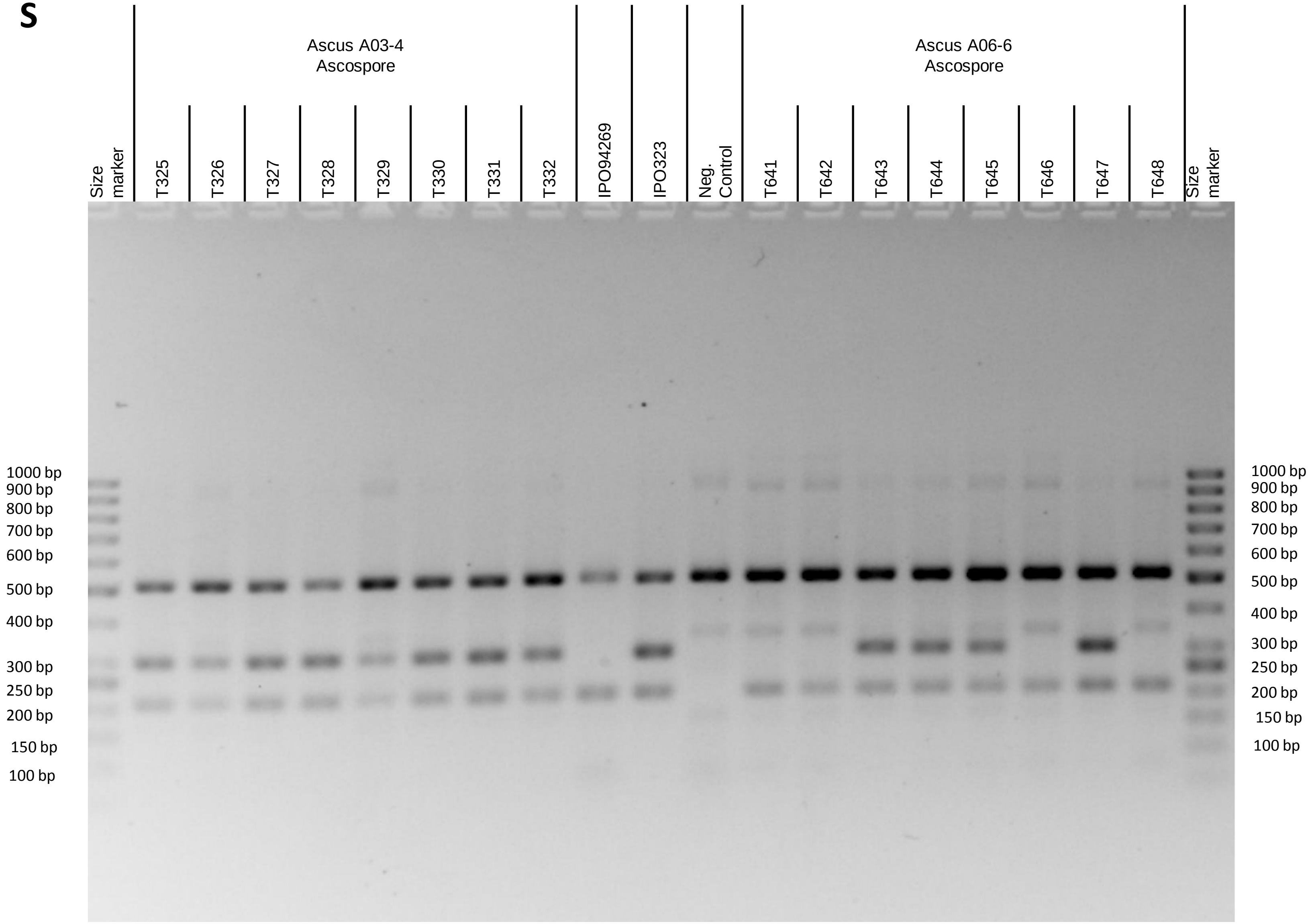

chr	Region	Primer-No	expected product size in IPO323 [bp]	Expected product size in IPO94269 [bp]
20	Centromer	2904*2905	300	-
2		879*880 (GAPDH)	207	207

T

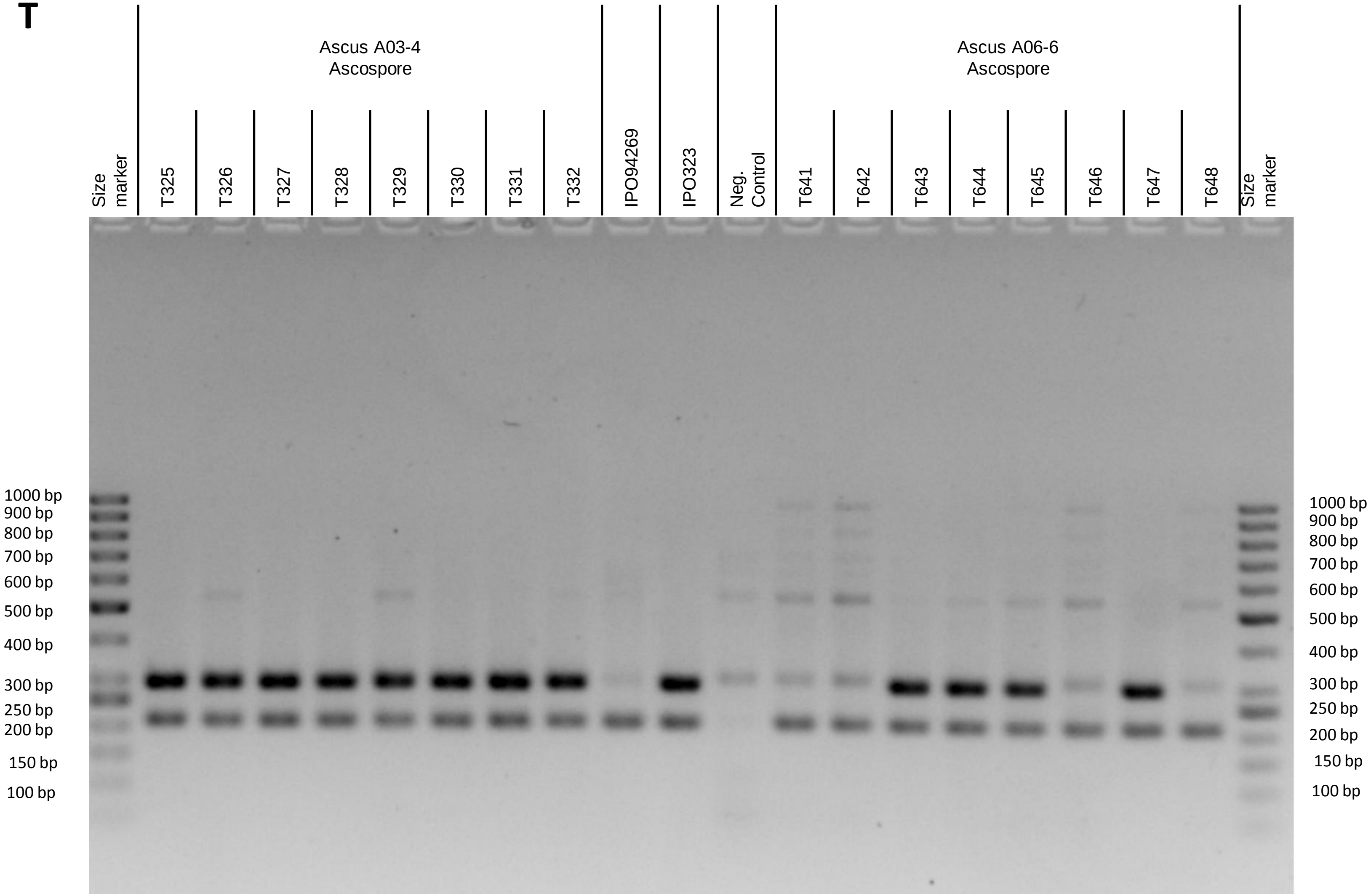

chr	Region	Primer-No	expected product size in IPO323 [bp]	Expected product size in IPO94269 [bp]
20	Subtelo mericR	2110*2111	300	-
2		879*880 (GAPDH)	207	207
